# Quantitative Microscopy Reveals Stepwise Alteration of Chromatin Structure during Herpesvirus Infection

**DOI:** 10.3390/v11100935

**Published:** 2019-10-11

**Authors:** Vesa Aho, Elina Mäntylä, Axel Ekman, Satu Hakanen, Salla Mattola, Jian-Hua Chen, Venera Weinhardt, Visa Ruokolainen, Beate Sodeik, Carolyn Larabell, Maija Vihinen-Ranta

**Affiliations:** 1 Department of Biological and Environmental Science and Nanoscience Center, P.O. Box 35, University of Jyvaskyla, 40014 Jyvaskyla, Finland; vesa.p.aho@jyu.fi (V.A.); elina.mantyla@tuni.fi (E.M.); satu.a.hakanen@jyu.fi (S.H.); salla.m.mattola@jyu.fi (S.M.); visa.ruokolainen@jyu.fi (V.R.); 2 BioMediTech, Faculty of Medicine and Health Technology, Tampere University, 33014 Tampere, Finland; 3 Molecular Biophysics and Integrated Bioimaging Division, Lawrence Berkeley National Laboratory, Berkeley, CA 94720, USA; axel.a.ekman@gmail.com (A.E.); jian-huachen@lbl.gov (J.-H.C.); vweinhardt@lbl.gov (V.W.); carolyn.larabell@ucsf.edu (C.L.); 4 Institute of Virology, Hannover Medical School, 30625 Hannover, Germany; sodeik.beate@mh-hannover.de; 5 Department of Anatomy, University of California San Francisco, San Francisco, CA 94143, USA

**Keywords:** HSV-1, chromatin, nuclear egress

## Abstract

During lytic herpes simplex virus 1 (HSV-1) infection, the expansion of the viral replication compartments leads to an enrichment of the host chromatin in the peripheral nucleoplasm. We have shown previously that HSV-1 infection induces the formation of channels through the compacted peripheral chromatin. Here, we used three-dimensional confocal and expansion microscopy, soft X-ray tomography, electron microscopy, and random walk simulations to analyze the kinetics of host chromatin redistribution and capsid localization relative to their egress site at the nuclear envelope. Our data demonstrated a gradual increase in chromatin marginalization, and the kinetics of chromatin smoothening around the viral replication compartments correlated with their expansion. We also observed a gradual transfer of capsids to the nuclear envelope. Later in the infection, random walk modeling indicated a gradually faster transport of capsids to the nuclear envelope that correlated with an increase in the interchromatin channels in the nuclear periphery. Our study reveals a stepwise and time-dependent mechanism of herpesvirus nuclear egress, in which progeny viral capsids approach the egress sites at the nuclear envelope via interchromatin spaces.

## 1. Introduction

Herpesvirus capsids assemble in the nucleus. During infection the formation of viral replication compartments (VRCs) induce extensive morphological reorganization of nuclear structures including chromatin marginalization [1,2,3,4,5]. Later in infection, progeny capsids translocate through the chromatin to their egress site at the nuclear envelope (NE). We have shown earlier that, late in infection, herpes simplex virus 1 (HSV-1) induces channels penetrating the layer of peripheral host chromatin [4], and we verified by mathematical modeling that such channels are sufficient for an efficient diffusion of capsids to the NE [6]. To obtain greater insight into the dynamics of HSV-1-induced reconstruction of nuclear architecture, it is important to determine the kinetics of chromatin remodeling and viral capsid mobility through the chromatin layer.

During lytic HSV-1 infection, the release of viral DNA into the nucleus is followed by a stepwise expression of viral genes, replication of viral DNA, and nuclear accumulation of viral proteins that lead to the formation of VRCs [7,8,9,10,11]. Initially several small VRCs appear that coalesce into an enlarged VRC, where the viral DNA is replicated and packaged into preassembled capsids [12,13]. At the last nuclear step of infection, progeny capsids translocate to the NE and egress by budding through the inner nuclear membrane, subsequently losing their transient primary envelope by fusing with the outer nuclear membrane so that non-enveloped capsids are released into the cytosol [14,15,16,17,18].

One of the first roadblocks on the viral egress pathway is the chromatin network that restricts the intranuclear movement of capsid-sized particles [6,19]. During HSV-1 infection, the formation of VRCs is accompanied by profound modifications of the chromatin architecture, including its marginalization and compaction to the nuclear periphery [3,6,11]. Chromatin marginalization does not only occur during herpesvirus infection, but also upon the infection of other DNA viruses, such as baculo- and parvoviruses [20,21,22]. The chromatin and, in particular, the compacted chromatin at the nuclear periphery constitutes a profound barrier for progeny viruses that need to leave the nucleus. Although proteins can often diffuse relatively freely in the nucleus [23], larger entities, such as intranuclear RNA and HSV-1 capsids, need to utilize interchromatin pathways intertwined between compact chromatin domains [19,24,25,26,27]. The motion of viruses in the nucleus is typically passive. The only known exception is baculovirus, which uses actin-dependent motility not only in the cytoplasm, but also in the nucleoplasm [28,29]. A similar mechanism has been suggested for the intranuclear movement of HSV-1 capsids [30,31]. However, recent studies indicated that the capsids move inside the nucleus by diffusion [19,32,33]. HSV-1 capsid passage through chromatin appears to depend on the presence of virus-induced interchromatin channels and enlarged interchromatin domains [4,19].

The key questions in understanding the nuclear egress of herpesviruses is how the chromatin architecture is altered during the infection, and how these changes enable the capsids to reach the NE. To answer these questions, we investigated the temporal changes of chromatin organization using soft X-ray tomography (SXT). Next, to gain insight into the spatiotemporal localization of capsids and chromatin, laser scanning confocal microscopy, expansion microscopy, and transmission electron microscopy imaging were used. Finally, to study capsid motion to the NE during the infection, we used the random walk modeling of capsid diffusion within the interchromatin networks reconstructed from the expansion microscopy images. Our studies reveal that the gradual enlargement of the VRCs correlates with a spatial reorganization and smoothening of the chromatin-VRC interface. At the same time, capsids are transported from the central VRCs towards the peripheral NE. Our modeling indicates that capsid translocation to the NE is slowed down during chromatin condensation and marginalization. Later, when interchromatin channels across the chromatin appear, capsid transport through the chromatin is accelerated. Our model suggests that the kinetics of capsid transport towards the NE correlated with the alterations in the chromatin architecture and indicate how virus-induced interchromatin channels support the nuclear egress of HSV-1.

## 2. Materials and Methods

### 2.1. Cells and Viruses

African green monkey kidney cells (Vero, ATCC® CCL-81™) were grown in Dulbecco’s modified Eagle medium (DMEM) supplemented with 10% fetal bovine serum, l-glutamine and penicillin-streptomycin (Gibco-Invitrogen, Carlsbad, CA, USA) at 37 °C in the presence of 5% CO_2_. The Epstein–Barr virus-transformed human B lymphocytes (GM12878) were purchased from the NGIMS Human Genetics Cell Repository, Coriell Institute of Medical Research (Camden, NJ, USA). B cells were maintained as suspension cultures at 37 °C in RPMI-1640 medium supplemented with 15% of fetal bovine serum (FBS), l-glutamine, penicillin, streptomycin (Gibco-Invitrogen), and 5% CO2. The medium was refreshed every 2–3 days to maintain a cell density of 0.8–2.0 × 10^6^  cells/mL. The HSV-1 strains used were wild-type 17+ strain, and EYFP-ICP4 (vEYFP-ICP4) [34], which were generous gifts from R. Everett (MRC Virology Unit, Glasgow, Scotland, UK). The viruses were amplified as previously described [34,35,36]. The cells were inoculated with wild-type HSV-1 17+ or HSV-1 EYFP-ICP4 at a multiplicitity of infection (MOI) of 5 and kept at 37 °C until live-cell microscopy or fixation.

### 2.2. Live Cell Imaging

Vero and human B cells were cultivated on 3 cm glass-bottom dishes (0.16 to 0.19 mm thickness; MatTek Cultureware, Ashland, MA, USA). Time-lapse imaging of the appearance and enlargement of VRCs was performed on Vero cells stained with Hoechst 33342 dye and on H2B-ECFP-expressing human B cells infected with EYFP-ICP4 HSV-1 at an MOI of 5. H2B-ECFP was a generous gift from Jörg Langowski (German Cancer Research Center, Heidelberg, Germany). The cells were imaged using Nikon A1R confocal microscope (Nikon Instruments inc., Tokyo, Japan) equipped with NIS Elements confocal imaging software. Prior to imaging, the medium was changed at 1.5 hpi, and the cells were allowed to stabilize for 30 min in the pre-heated microscope incubator set to 37 °C. The specimen environment was maintained at 37 °C with the CO_2_ concentration at 5% during microscopy. Hoechst, EYFP, or ECFP were excited with a 405 nm diode laser, a 488 nm argon laser line, and a 458 nm argon laser line, respectively. Fluorescence was detected with a 450/50 nm, a 515/30 nm, or a 482/35 nm band-pass filters. CFI Plan Apo VC 60XC objective lens was used during imaging (NA 1.2, WD 0.31–0.28, water immersion). Images of Vero and human B cells were taken at 10 and 5 min intervals, respectively.

### 2.3. Soft X-ray Tomography

Human B cells infected with vEYFP-ICP4 were collected by centrifugation (125× *g*, 10 min) at 8, 12, 16, and 20 hpi. The B cells were used for the SXT image acquisition because of their HSV-1 susceptibility and small size, allowing their placement into thin-walled capillaries of a diameter of up to 15 µm [4,37,38]. The cell pellet was resuspended in Leibovitz’s L-15 medium without phenol red (Gibco-Invitrogen) supplemented with 15% of FBS (ATCC, Manassas, VA). The infected cells were separated from non-infected cells using fluorescence-activated cell sorting (FACS, BD FACSAria, BD Biosciences, San Jose, CA, USA) by gating for the expression of EYFP. The cells with small nuclear EYFP-ICP4 foci at 8 hpi and cells with single enlarged VRC at 12, 16, and 20 hpi were selected. To reduce the residence time of specimens in FACS sheath fluid (PBS 1×), the cells were sorted directly into L-15 with 15% FBS growth medium. The sorted, infected cells were prepared and frozen into glass capillaries as described in our earlier work [4]. Imaging was done using a XM-2 soft X-ray microscope in the National Center for X-ray Tomography located at the Advanced Light Source of the Lawrence Berkeley National Laboratory. Capillaries were kept in a stream of liquid nitrogen-cooled helium gas during data collection [39,40]. Each dataset contained 90 projection images collected sequentially around the axis of rotation in 2° increments, which gives a total rotation of 180°, using a 300–400 ms exposure time. The voxel size was 35 nm. A series of 10 reference images were taken before and after the scan to normalize the data [41]. The projection images were aligned and reconstructed using automated registration software AREC3D [41].

The nuclei of the cells were segmented in the tomographic reconstructions by hand using the AMIRA software package. Segmentation of the nuclei into high- and low-density regions was performed using the Gaussian mixture model (GMM) [42]. After performing the GMM fitting, we extracted probability maps of the two regions. These maps were then smoothed using a Gaussian kernel of σ = 1 px before predicting the labels, which removes substantial amount of noise in the classification.

To clean up the binarization, the holes of the low-density VRC region were filled, and isolated regions of VRC removed (these can be found mostly outside the nuclear envelope from possible mis-segmentation or variation of linear absorption coefficient). Error estimates of the results were obtained by varying the threshold probability for the GMM by ±10%. To measure the roughness of the VRC-chromatin boundary, we determined the surface-area-to-volume ratio, SA/V, of the boundary. Surface-area-to-volume ratio is a common metric describing the morphology of cells. To incorporate the varying shape of the nucleus itself, SA/V was normalized with the SA/V of the nuclear envelope. The surface area was measured using the marching cubes algorithm [43]. The uncertainties of the individual data points were used to calculate the weighted averages and confidence intervals of SA/V.

### 2.4. Immunofluorescence Microscopy

Vero cells were cultivated on cover glasses (0.17 mm thickness, 18 × 18 mm², Carl Zeiss, Jena, Germany) and infected with the wild type HSV-1 strain 17+ at an MOI of 5, and fixed in 4% paraformaldehyde in phosphate-buffered saline for 12 min at room temperature. Cell samples were incubated with a primary mouse antibody against HSV-1 capsid protein VP5 (mAb 6F10, Santa Cruz Biotechnology Inc., Dallas, TX, USA), and with a mixture of primary rabbit antibodies against histone modifications H3K27ac (Abcam 4729, Abcam, Cambridge, UK), H3K9me3 (NB211073, Novus biological, Cambridge, UK) and H4K20me3 (Abcam 9053) to label chromatin. The primary antibodies were followed by secondary Alexa 488-conjugated goat anti-mouse and Alexa 555-conjugated goat anti-rabbit antibodies (Thermo Fisher Scientific, Waltham, MA, USA). Unexpanded samples were mounted with ProLong Glass Antifade Mountant with NucBlue (Thermo Fisher Scientific). For expansion samples, a threefold concentration of primary antibodies and a fourfold concentration of secondary antibodies were used to retain the signal after expansion. Sample expansion was performed using the previously described proExM Protocol for Cultured Cells, V1.0 [44]. Alexa 488 and Alexa 555 were excited with a 488 nm argon laser and a 561 nm sapphire laser, respectively. Fluorescence was detected with a 515/30 nm, and a 595/50 band-pass filters. For unexpanded samples, CFI Plan Apochromat VC 60× objective lens (N.A 1.4, WD 0.13, oil immersion) was used during imaging, and stacks of 512 × 512 pixels were collected with a pixel size of 60 nm in the x- and y-directions and 150 nm in the z-direction. Image analysis was done with ImageJ [45]. For expansion microscopy sample imaging, CFI Plan Apochromat VC 60X (N.A 1.2, WD 0.31–0.28, water immersion) lens was used. Stacks of 1024 × 1024 pixels were collected with a pixel size of 90 nm in the x- and y- directions, and 180 nm in the z-direction. To quantify the intensity of fluorescent labels in the expanded samples as a function of the distance from the nuclear envelope, the nucleus was segmented by using minimum cross entropy segmentation of the stained chromatin signal [46] and by filling possible holes within the segmented geometry. The nuclear envelope was defined by the pixels lying immediately outside the segmented nucleus. The 3D Euclidean distance of every point in the nucleus to the NE was calculated, and the pixels were then sorted into 180 nm (corresponding to ~45 nm distance due to the expansion) wide bins based on their distance to the NE. The average fluorescence intensity for each bin was calculated.

### 2.5. Transmission Electron Microscopy

Vero cells infected with wild type HSV-1 strain 17+ (MOI 5) and non-infected control cells were fixed in 3.9% paraformaldehyde and 0.25% glutaraldehyde in 50 mM phosphate buffer (at a pH of 6.8) followed by post-fixation in 1% OsO_4_ for 1 h on ice. Cells were dehydrated with ethanol and then embedded in low viscosity embedding resin (TAAB Laboratories Equipment Ltd, UK). Thin sections were cut with an Ultracut UC6a ultramicrotome (Leica Mikrosysteme GmbH, Germany) and collected on Pioloform-coated, single-slot copper grids. Sections near the middle plane of the nucleus were selected, and they were stained with 2% aqueous uranyl acetate and lead citrate and examined with a JEOL JEM-1400 electron microscope (JEOL Ltd., Tokyo, Japan) operated at 80 kV. The images were captured using a bottom mounted Quemesa CCD camera with a resolution of 4008 × 2664 pixels (EMSIS GmbH, Münster Germany).

### 2.6. Numerical Simulations of Capsid Motion

To simulate HSV-1 capsid movement in cells, the nuclei were segmented into chromatin and interchromatin regions by calculating minimum cross entropy threshold [46] of the stained chromatin using only the pixels that were inside the nucleus. The capsids were allowed to travel within the interchromatin domains, and the diameter of the capsids was set to 125 nm. The diffusion was modeled using a fixed step (~11 nm) random walk algorithm, and the number of simulated particles in each cell geometry was 1000. The initial location of each capsid was randomly selected from the interchromatin region at a distance further than 1 µm from the NE.

## 3. Results

### 3.1. Formation of Nuclear Replication Compartments

**Figure 1:**
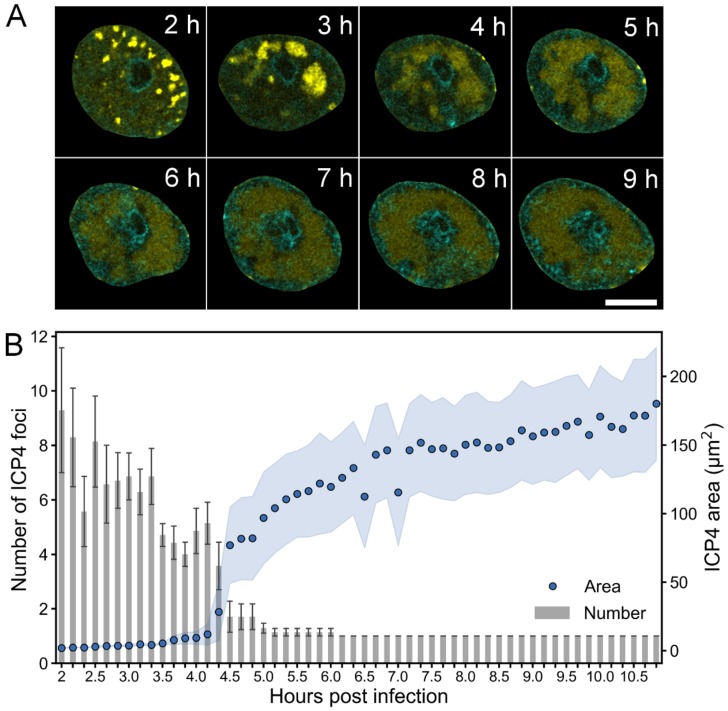
Formation of HSV-1 replication compartment. (**A**) Confocal microscopy images showing the distribution of EYFP-ICP4 (yellow) at 2–9 hpi in Vero cells with DNA visualized using Hoechst 33342 dye (cyan). Scale bar is 5 µm. See also Movie S1 and Figure S1. (**B**) A quantitative analysis of the number and area of EYFP-ICP4 foci during infection (*n* = 7). The mean values of the number of EYFP-ICP4 foci ± the standard error of the mean (SEM) are shown. The blue shading around the area data points represent the mean ± SEM.

To follow the formation of the HSV-1 VRCs and the changes in host chromatin over time, we performed time-lapse fluorescence confocal microscopy of infected Vero cells and examined the subcellular localization of the immediate early protein ICP4, a major viral transactivator that is known to localize to VRCs [47]. We studied the distribution of ICP4 and Hoechst 33342-labeled DNA in cells infected with EYFP-ICP4 HSV-1 [34]. At 2 hpi, EYFP-ICP4 was located in nuclear foci in a distinct punctate pattern, and around 4 hpi a larger VRC formed as smaller individual VRCs coalesced (Figure 1A, Movie S1). While the number of VRC foci decreased, their size increased (Figure 1B). Expansion of the VRCs was accompanied by a relocation of the host chromatin to the nuclear periphery. The enlarged VRCs covered most of the nucleus but excluded the nucleolus. Next, we analyzed the infection in human B cells, which are also susceptible to HSV-1 [4] and sufficiently small for SXT sample capillars. The low penetration depth of soft x-rays limits the capillar diameter to about 15 µm, which is too small for SXT analyses of Vero cells. Single cell analyses showed that enlarged VRCs formed later in human B cells (after 7.5 hpi, Figure S2, Movie S2) than in Vero cells (after 4 hpi). The average volume occupied by EYFP-ICP4 increased from 10 to 110 µm^3^ between 6 and 11.5 hpi. While the enlarged VRCs formed, histone H2B-ECFP, a marker of chromatin, was displaced from the EYFP-ICP4 positive regions, forming a layer at the nuclear periphery.

These experiments showed that the formation of a single enlarged VRC and chromatin marginalization are concurrent processes during HSV-1 infection.

### 3.2. Infection-Induced Nuclear Swelling and Chromatin Straightening

**Figure 2:**
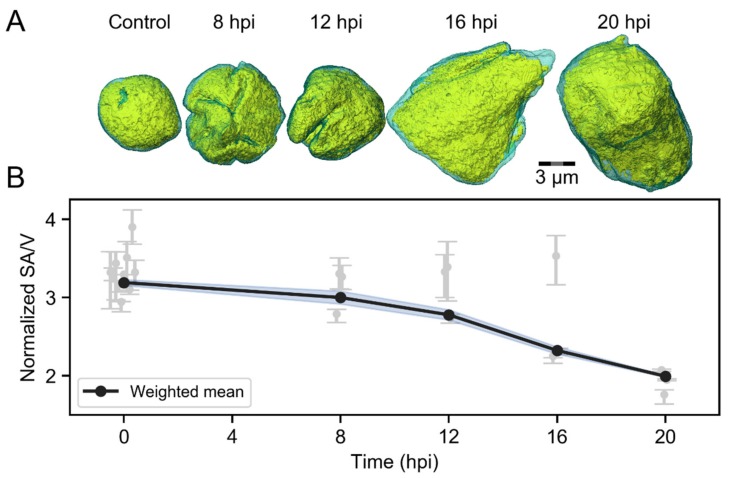
Infection-induced changes in the viral replication compartment (VRC) volume and chromatin smoothening. SXT analyses of non-infected (*n* = 10) and infected (*n* = 3) human B cells at 8, 12, 16 and 20 hpi. (**A**) Volume-rendered 3D views of nuclei showing high-density regions (condensed chromatin) in cyan and low-density regions (VRC and low-density chromatin) in yellow. Scale bar is 3 µm. (**B**) An analysis of SXT images, showing the effect of VRC enlargement on the folding of marginalized chromatin. The error-weighted averages of normalized surface area to volume ratios (SA/V) of chromatin layer around the VRC are shown. The shaded areas around the data points represent the standard error of the weighted mean (blue).

To study how the nuclear accumulation of viral DNA and proteins affects the chromatin layer surrounding the VRCs, we measured the changes in VRC volume and smoothening of the chromatin-VRC interface by SXT analyses of infected human B cells (Figure 2A). SXT allows us to visualize nuclear compartments, such as chromatin and VRC, in high resolution [4,6]. At 8 hpi, the VRC volume was similar (251 ± 12 µm^3^, *n* = 3) to the euchromatin area of non-infected cells (230 ± 80 µm^3^, *n* = 10). Later in infection, the VRC volume increased to 500 ± 200 µm^3^ at 12 hpi, 500 ± 100 µm^3^ at 16 hpi, and 600 ± 300 µm^3^ at 20 hpi (*n* = 3), respectively (Figure 2B). We determined the smoothness of the chromatin-VRC interface by analyzing the ratio of surface area to volume (SA/V) surrounding the VRCs. The weighted averages of normalized SA/V at 8 and 12 hpi, 4.0 ± 0.2 and 3.56 ± 0.13, were similar to the SA/V of the heterochromatin layer surrounding the euchromatin in non-infected cells of 4.38 ± 0.08. During infection, the surface to volume ratio decreased to 2.6 ± 0.1 at 16 hpi and further to 1.99 ± 0.03 at 20 hpi. Our data suggest that the expansion of the VRCs might induce smoothening of the chromatin layer as the nucleus enlarges.

### 3.3. Virus-Induced Changes in Chromatin Distribution and Nuclear Volume

To further analyze the infection-induced changes in chromatin, we used protein retention expansion microscopy (proExM) [44] to get a fourfold increase in the size of infected Vero cells, which consequently increases the resolution of microscopy images by the same factor. ProExM is based on embedding a fixed and fluorescently labeled sample into a swellable polymer matrix. The matrix is then immersed in water, which causes it to expand isotropically, increasing the size of the sample and the distance between the fluorophores. When the fluorescent labels move further away from each other during the expansion, their probability to be resolved separately increases. Based on their size, HSV-1 capsids can travel through interchromatin channels that are as small as 125 nm in diameter, which means that they cannot be resolved using conventional light microscopy. With the fourfold increase in resolution, these channels can be visualized with proExM. This is particularly important for the simulations of capsid transport through the chromatin that we describe later in this paper.

**Figure 3:**
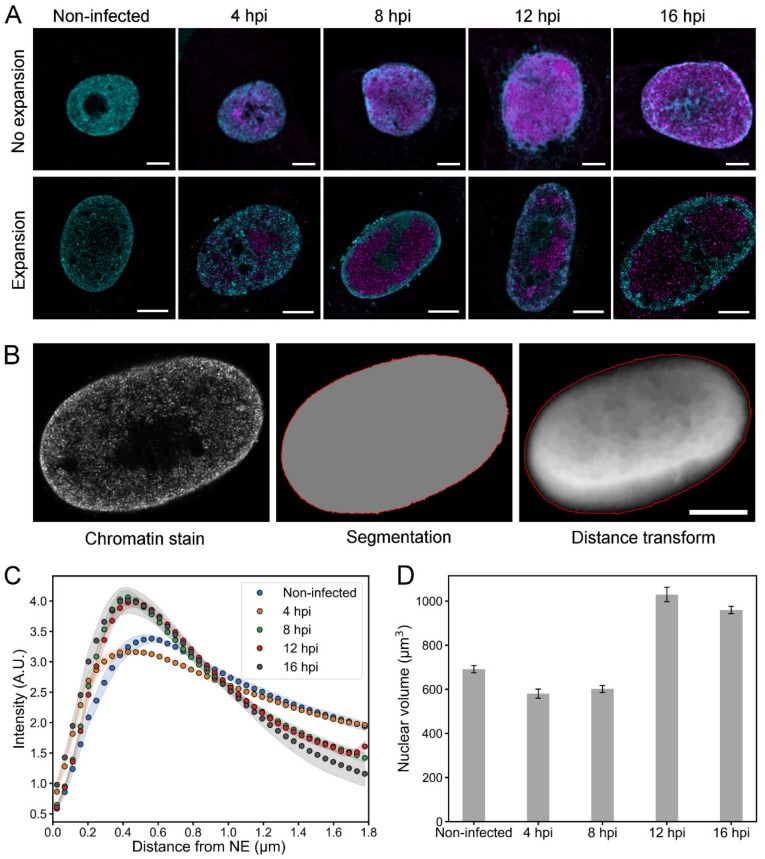
Chromatin distribution and nuclear volume of infected Vero cells. (**A**) Confocal microscopy slices of unexpanded and expanded nuclei of non-infected and infected cells at 4, 8, 12, and 16 hpi. Chromatin was stained with a mixture of histone Abs (H3K27ac, H3K9me3, H4K20me3; cyan) and viral capsid protein with VP5 MAb (magenta). Scale bars are 5 µm. (**B**) A set of images showing a segmented nucleus and the positioning of its nuclear envelope (red) based on the chromatin staining. A distance transform, showing the Euclidean distance to the NE, is also shown. The intensity of each pixel is proportional to the distance to the NE. Note that in the central parts of the nucleus the main contribution to the distance values result from the distance to the basal and apical surfaces of the nucleus, which are not shown in the image. Scale bar is 5 µm. (**C**) A quantitative analysis of the relative amount of chromatin in 3D expansion microscopy images of non-infected (*n* = 9) and infected (*n* = 9–10) cells as a function of the distance from the NE. The shaded areas around the data points represent the mean ± SEM. (**D**) The nuclear volume at various times after infection analyzed from 3D confocal images. The mean nuclear volume ± SEM is shown.

Consistently with unexpanded cells, proExM images showed that the chromatin markers were concentrated in the nuclear periphery at 8, 12, and 16 hpi (Figure 3A). At 4 hpi and in non-infected cells, the chromatin was located both in the nuclear periphery and in the central nucleoplasm. The nuclei of the cells were segmented based on the chromatin staining, and the distance of each nuclear location to the NE was determined (Figure 3B). A quantitative analysis of the chromatin as a function of distance from the NE showed that in infected and non-infected cells the amount of chromatin was highest near the NE (Figure 3C). In infected cells, chromatin accumulated in the nucleoplasm about 400 nm away from the NE, while in non-infected cells its maximum was at around 550 nm from the NE. A quantitative 3D analysis showed that the average volumes of the nuclei at 4 and 8 hpi (580 ± 70 μm^3^, *n* = 10; 600 ± 50 μm^3^, *n* = 10) were similar to that of non-infected cell nuclei (690 ± 50 μm^3^, *n* = 10) (Figure 3D). At 12 and 16 hpi, the nuclear volume had increased to 1030 ± 110 μm^3^ (*n* = 11) and 960 ± 50 μm^3^ (*n* = 10), respectively. Notably, in human B cells the nuclear enlargement occurred later at 16 hpi (Figure 2). This likely reflects cell type-dependent differences in the progress of HSV-1 infection (Figure 1 and Figure S2).

Altogether, our results show quantitatively the relocalization of the host chromatin toward the nuclear periphery and the subsequent enlargement of nuclear volume later in infection.

### 3.4. Intranuclear Distribution of Capsid Proteins and Capsids

**Figure 4:**
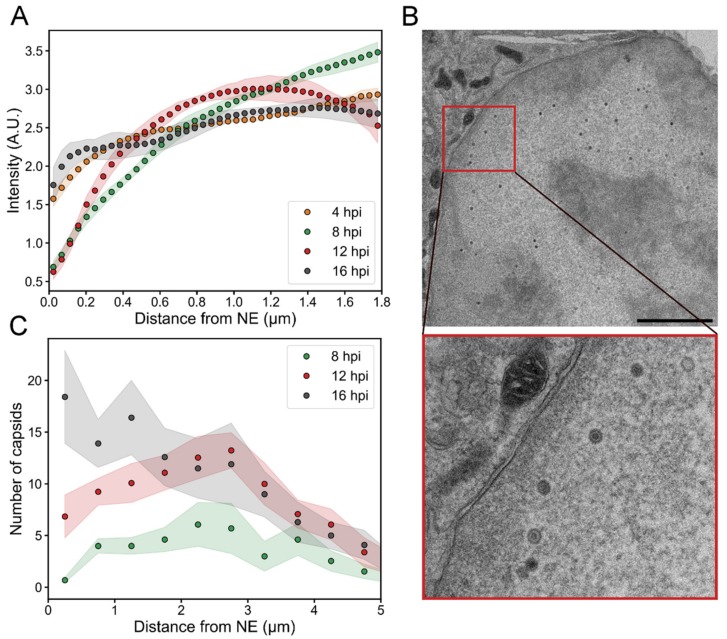
Viral capsid and capsid protein localization in the nucleus. The distribution of the VP5 capsid protein and capsids as a function of the distance from the NE at various times post-infection. (**A**) A quantitative analysis of 3D confocal expansion microscopy images of infected cells (*n* = 9–10) showing the localization of VP5 as a function of the distance from the NE. (**B**) A TEM image of an infected cell at 16 hpi. The inset shows an enlarged view of the boxed area containing capsids in the proximity of the NE. Scale bar is 3 µm. (**C**) An analysis of TEM images illustrating the number of capsids as a function of the distance from the inner nuclear membrane (*n* = 10–13). The shaded areas around the lines represent the mean ± SEM.

We used proExM to follow the intranuclear distribution of HSV-1 capsid protein VP5 during the infection. The quantitative analysis of the relative amount of VP5 as a function of the distance from the NE indicated that at 4 hpi VP5 was distributed in the nucleus quite uniformly (Figure 4A). When the infection proceeded, VP5 relocated from the nuclear periphery towards the center. Later, at 12 hpi, part of the VP5 signal had shifted back from the central parts of the nucleus towards the NE, but the amount of VP5 next to the NE remained low. Finally, at 16 hpi, we detected an increased presence of VP5 in close proximity to the NE.

To further study the capsid localization inside the nucleus, we analyzed transmission electron microscopy images of infected cells. Since the partial heterogeneity of cell slices did not allow accurate segregation between empty and DNA-containing capsids, both types of capsids were taken into account. This approach is also justified by the fact that the diffusion of empty and full capsids is not expected to differ within the nucleus since they are of the same size [48]. The analysis showed an increase in the number of capsids close to the NE (Figure 4B,C). The average number of nuclear capsids at 8, 12 and 16 hpi per nuclear cross-section was 38 ± 12, 93 ± 11, and 120 ± 30 (*n* = 13, 13, 10), respectively. At 4 hpi, we did not detect any capsids. At 8 hpi, the capsids were located quite uniformly inside the nucleus, but the average number within 500 nm from the NE was low (0.7 ± 0.3). At 12 hpi, there was a clear increase in the number of capsids near the NE (7 ± 2), and at 16 hpi the capsid distribution was even more peripheral (18 ± 4 capsids within 500 nm from the NE).

In summary, our results show that the displacement of VP5 from the peripheral nucleoplasm towards the center of the nucleus correlated temporally with the appearance of capsids in the center of the nucleus between 4 and 8 hpi. The data also shows the gradual increase of capsids near the nuclear envelope during the infection.

### 3.5. Simulations of the Nuclear Transport of HSV-1 Capsids

**Figure 5:**
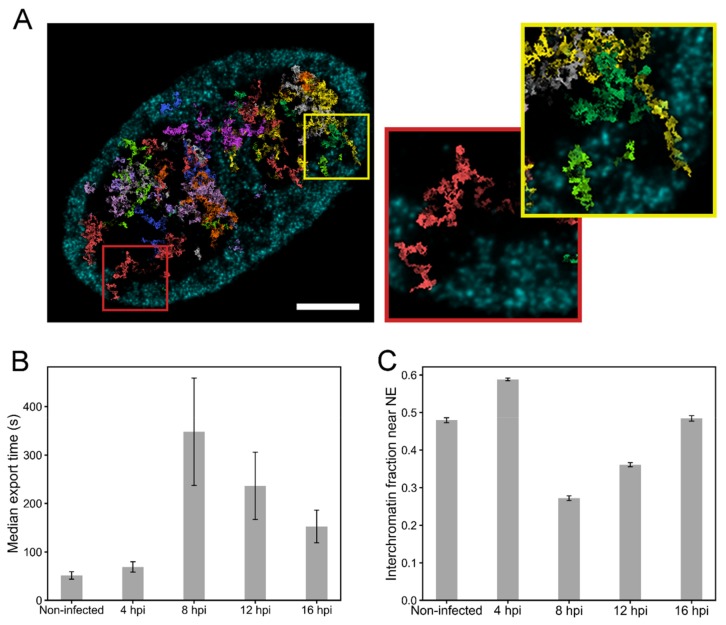
Nuclear transport of capsids through the chromatin. (**A**) Simulated random walk tracks of 40 capsids in the chromatin reconstruction of an expansion microscopy image at 16 hpi, recorded in a 450 nm thick cell volume. Ten distinct colors are used to separate the paths drawn by the capsids. The maximum intensity projection of chromatin in the region is shown in cyan. Scale bar, 5 µm. The magnified pictures show the paths of two capsids reaching the NE through the interchromatin space. See also Movie S3. (**B**) The simulated median transport times of capsids to the NE in the non-infected (*n* = 9) and infected (*n* = 9–10) cells. (**C**) The ratio of interchromatin volume to the total volume in the region located from 250 nm to 750 nm from the NE. The error bars show the mean ± SEM.

To assess how the changes in the chromatin structure during infection affect the subnuclear transport of capsids, we simulated their motion towards the NE using a simple random walk model where the capsids were allowed to travel in the interchromatin regions and the motion into the chromatin regions was prevented [6]. The model was used to simulate paths of HSV-1-sized particles (125 nm) in 3D reconstructions of chromatin generated from the proExM images (Figure 5A, Movie S3). We segmented nuclei into chromatin and interchromatin regions, and modeled particles with a diffusion coefficient of 2.2 × 10^−2^ µm^2^/s [19] in the interchromatin regions. Since the chromatin dynamics in cells, especially during virus infection, are not known, we assumed stationary chromatin structure. In every cell geometry, we simulated diffusion of 1000 non-interacting particles. The simulations revealed that capsids were able to reach the NE through the low-density channels across the host chromatin. We measured the transport time of each capsid to the NE, and recorded the median transport time for each cell (Figure 5B). The transport time of capsids to the NE was relatively short in control cells and after 4 hpi. At 8 hpi, when the VRCs had enlarged and the marginalized chromatin formed a permeability barrier, the capsid transport time to the NE increased. At 12 and 16 hpi, the transport time to the NE decreased again. One likely explanation for the faster transport times at 12 and 16 hpi is the formation of interchromatin channels in the marginalized chromatin [4]. To test this hypothesis, we analyzed the fraction of interchromatin volume within the marginalized chromatin layer from the expansion data (Figure 5C). In the range of 250–750 nm from the NE, the interchromatin fraction was high in non-infected cells and at 4 hpi, but at 8 hpi the interchromatin fraction had decreased. However, at 12 hpi and 16 hpi, the interchromatin fraction increased again. These results suggest that interchromatin space was created in the marginalized chromatin layer to permit a more efficient capsid transport to the NE.

## 4. Discussion

**Figure 6:**
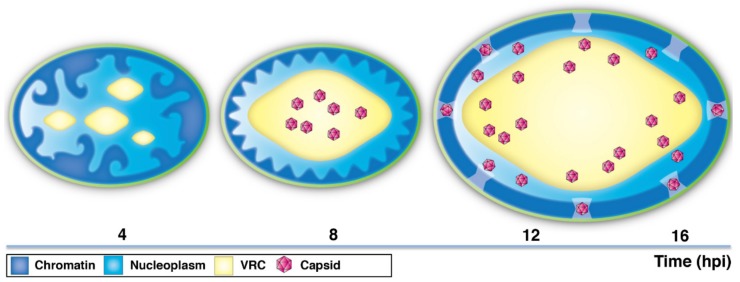
A schematic presentation of the time-dependent changes in chromatin architecture and distribution of HSV-1 capsids. At early infection (~4 hpi), the viral replication compartments (VRCs) appear as distinct nuclear foci. Later in infection (~8 hpi), small VRSs undergo fusion and expansion into a large, globular VRC. The chromatin is marginalized and compacted close to the NE. The capsids are mostly located in the central regions of the nucleus. At late infection (12–16 hpi), the enlargement of the nucleus and the VRC is accompanied by smoothening of the chromatin layer and increase in the interchromatin space in the nuclear periphery. The capsids travel towards the peripheral nucleoplasm and the NE.

Profound modifications of the host chromatin architecture occur during lytic herpesvirus infection. By combining state-of-the-art imaging techniques with advanced image analysis and numerical modeling, we investigated the timing of virus-induced reorganization of chromatin and the gradual movement of virus capsids through the interchromatin space towards the NE (Figure 6).

Our studies demonstrated that the growth of enlarged VRCs in the nucleoplasm is accompanied by a spatial reorganization of host chromatin into the nuclear periphery. The changes in chromatin localization occurred concurrently with the growth of VRCs from several smaller foci to a large globular VRC after 4 hpi. Later in infection, the volumes of the nuclei increased. The enlargement of the VRC contained within the marginalized chromatin layer is likely the driving force behind the smoothening of the chromatin layer that we observed. It can also be assumed that this smoothening is beneficial for capsid egress, since a smooth interface allows capsids to lie closer to the NE than a rough interface does. The time scale of the changes in the nuclear architecture depends on the virus. In parvovirus and baculovirus infection, VRC expansion and chromatin marginalization are detected at 20–24 hpi [49,50,51] or at 16–24 hpi, respectively [22]. The exact nature of the molecular mechanisms mediating the virus-induced chromatin marginalization and condensation is unknown. In non-infected cells, chromatin condensation to the nuclear periphery is a hallmark of preapoptotic cellular shrinkage [52,53,54]. Thus, it has been suggested that the nuclear appearance of naked herpesviral DNA and its interaction with the interferon-inducible protein 16 (IFI16) could trigger DNA sensor-mediated signal transduction leading to programmed cell death [55,56,57,58,59,60]. Furthermore, mechanical forces might also contribute to chromatin condensation, e.g., by depletion attraction forces and phase separation phenomena, which are induced by the expansion of VRCs into large globular structures [3,19,20,61].

Our proExM analysis combined with transmission electron microscopy showed that the capsid protein VP5 was displaced from the peripheral nucleoplasm concurrently with the appearance of viral capsids in the central nucleus between 4 and 8 hpi. This suggests that the amount of free VP5 in the nucleus is low at 8 hpi, and most of it is associated with the assembled capsids. The electron microscopy images showed that an increasing number of capsids moved closer to the NE at 16 hpi, suggesting that a large number of capsids reaches the NE for egress at this time. Although our results show that a large number of viruses reach the NE late in the infection, it seems likely that individual capsids might access the NE earlier. To analyze the timing of nuclear egress, high resolution time-lapse microscopy of single capsids during their passage through the chromatin is required.

Our mathematical modeling of capsid motion in the 3D reconstructions of chromatin network, obtained by expansion microscopy, revealed that the transport times to the NE vary during infection. The capsid transport rate was similar in the non-infected and 4 hpi chromatin networks, suggesting that initially the distribution and density of chromatin is relatively unaltered. Capsid transportation is slowed down when the host chromatin accumulates close to the NE. This suggests that the condensed host chromatin constitutes a barrier for the capsids on their way towards the NE. Notably, the transport of capsids seems to become accelerated later in infection concomitantly with the increase in the interchromatin space next to the NE. This is in line with recent studies demonstrating the herpesvirus-induced emergence of interchromatin domains and low-density channels in the chromatin layer [4,19]. In infected cells, the location of the interchromatin channels seems not to be correlated with the positions of the nuclear pore complexes [4] whereas in non-infected cells the channels are connected to nuclear pore complexes. These naturally occurring channels are used for the intranuclear transport of nuclear RNA and proteins [26,62,63,64,65,66,67,68,69]. Note that in our model the diffusion coefficient of the capsids in the interchromatin regions was assumed to stay constant during the infection. It is possible that the diffusion coefficient in late infection changes due to macromolecular crowding. Single particle tracking of virus capsids at various infection time points is needed to answer whether the diffusion coefficient changes, but the tracking at late infection is probably very challenging due to the increasing number of produced capsid proteins and assembled capsids.

Our studies provide the first indication that the transportation of HSV-1 capsids to their egress sites at the NE is correlated with stepwise modifications of the chromatin. Initially, the time-dependent compaction of the host chromatin, which is associated with an expansion of the VRCs, constitutes a barrier between capsids and their egress sites at the NE. Later, the smoothening of the chromatin-VRC interface and the increase of interchromatin space related to nuclear expansion results in enhanced capsid transport through the chromatin barrier. The role of viral proteins and their interactions with nuclear structures during chromatin remodeling remains to be elucidated. For a better understanding of virus egress strategies, it will be crucial to analyze single capsid dynamics and viral protein interactions in the chromatin network in more detail.

## Supplementary Materials

The following are available online at https://www.mdpi.com/1999-4915/11/10/935/s1, Figure S1: Live cell time-lapse imaging of chromatin marginalization in Vero cells, Figure S2: Live cell time-lapse imaging of the formation of HSV-1 replication compartment in B cells, Movie S1: Time-lapse imaging of VRC maturation in Vero cells, Movie S2: Time-lapse imaging of VRC maturation human B cells, and Movie S3: Simulated capsid paths.
