# Supplementary material for: Quantitative Microscopy Reveals Stepwise Alteration of Chromatin Structure during Herpesvirus Infection
Source: Viruses. 2019 Oct 11;11(10):935. doi: 10.3390/v11100935 (PMC6832731; doi:10.3390/v11100935)
Supplement: Supplementary file 1 [file viruses-11-00935-s001.zip › Captions of Supplementary Movies.pdf]

Movie S1. Time-lapse imaging of VRC maturation in Vero cells. The nuclear distribution of EYFP-ICP4 (yellow), known to localize to the nuclear VRCs, was visualized between 2 and 16 hpi. The localization of DNA was shown by Hoechst 33342 dye (cyan).

Movie S2. Time-lapse imaging of VRC maturation human B cells. The intranuclear distribution of EYFP-ICP4, a marker for VRC, was visualized between 6 and 11.5 hpi by using pseudocolor with intensity increasing from blue to white. The localization of chromatin was shown by histone H2B-ECFP (gray).

Movie S3. Simulated capsid paths. Tracks drawn by the motion of 40 particles during 95 seconds, shown in a 450 nm thick volume of the cell. The intensity of the path is a linearly decreasing function from the maximum value in the central plane to zero (black) in the top and bottom surfaces of the volume. The maximum intensity projection of the chromatin in the region is also shown (cyan).
