# Supplementary material for: Quantitative Microscopy Reveals Stepwise Alteration of Chromatin Structure during Herpesvirus Infection
Source: Viruses. 2019 Oct 11;11(10):935. doi: 10.3390/v11100935 (PMC6832731; doi:10.3390/v11100935)
Supplement: Supplementary file 1 [file viruses-11-00935-s001.zip › Supplementary Figures.pdf]

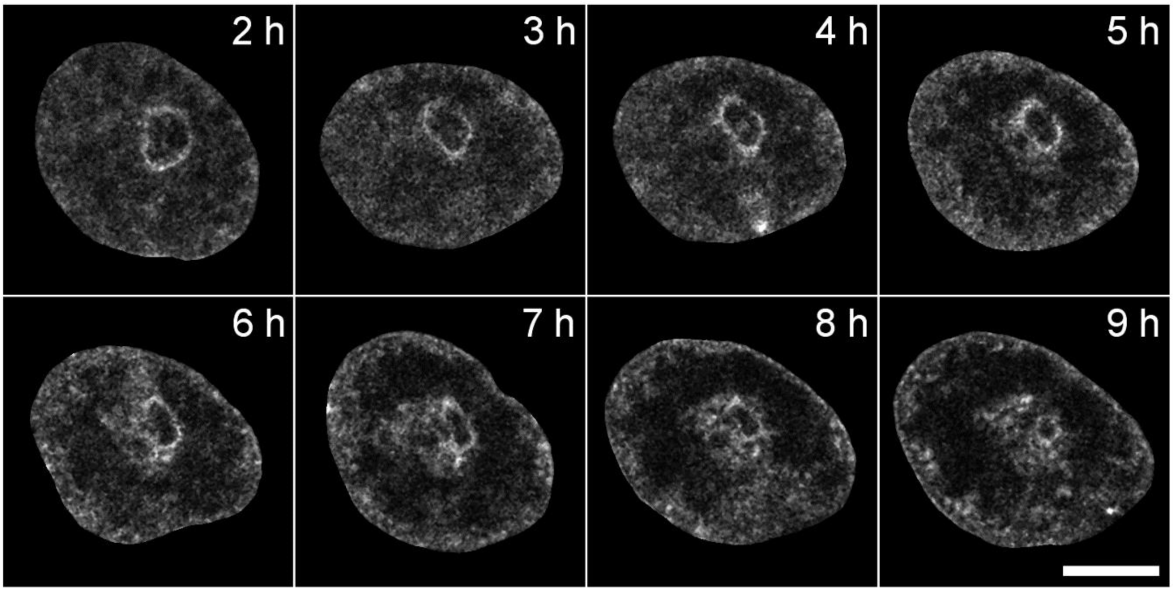

Figure S1. Marginalization of chromatin in a Vero cell during HSV-1 infection. Confocal microscopy images show the distribution of Hoechst 33342 dye at 2-9 hpi. The images were normalized to have the same average intensity. Scale bar is 5 μm.

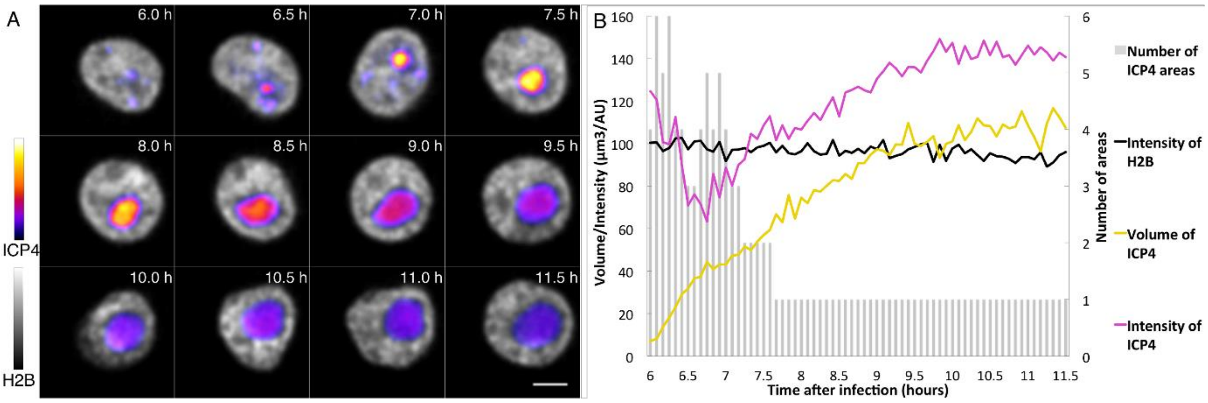

Figure S2. Live cell time-lapse imaging of the formation of HSV-1 replication compartment in B cells.
(A) Confocal microscopy images showing the phases of HSV-1 EYFP-ICP4 distribution at 6-11.5 hpi in a histone H2B-ECFP (grayscale) expressing cell. In order to emphasize changes in the fluorescence intensity, pseudo-color images with the intensity increasing from blue to white are shown. Scale bar is 3 μm. See also Movie S2.
(B) Quantitative analysis of the temporal changes in the number of EYFP-ICP4 regions, average fluorescence intensity and volume of EYFP-ICP4 regions together with the average fluorescence intensity of H2B-ECFP (n=6).
